# Alginate‐Based Microencapsulation of *Saccharomyces Boulardii* for Enhanced In Vitro Stability in Poultry Sector: A Novel Strategy for Probiotic Delivery

**DOI:** 10.1002/vms3.70841

**Published:** 2026-03-16

**Authors:** Elahe Khademi, Fahimeh Nemati, Tohid Piri‐Gharaghie

**Affiliations:** ^1^ Department of Biotechnology TeMS.C., Islamic Azad University Tehran Iran; ^2^ Biotechnology Research Center, ZistYar Sanat Technology Development Group Science and Technology Park, Tarbiat Modares University Tehran Iran

**Keywords:** alginate, feed additive, gastrointestinal stability, microencapsulation, poultry feed, *S. boulardii*, shelf‐life

## Abstract

**Background and Objective:**

*Saccharomyces boulardii* is a probiotic yeast with promising effects on poultry gut health and immunity, yet its application is limited by sensitivity to heat, pH and storage conditions. This study aimed to develop and evaluate an alginate‐based microencapsulation system to improve the in vitro stability, storage viability and antimicrobial performance of *S. boulardii* for poultry feed applications.

**Methods:**

A probiotic strain of *S. boulardii* was isolated, characterised and cultured under optimised fermentation conditions. The yeast was encapsulated using sodium alginate and cryoprotectants via extrusion, followed by freeze‐drying. Microcapsules were characterised using FESEM, DLS, zeta potential and FTIR. Probiotic traits, including acid and bile salt tolerance, NaCl resistance, antibiotic susceptibility and antagonistic activity against common pathogens, were assessed. Encapsulation efficiency, viability before/after processing and shelf‐life at 4°C and 25°C over 90 days were also evaluated.

**Results:**

The microencapsulation efficiency was 73.27% ± 0.39%, with stable spherical capsules averaging 2–5 µm in size. The encapsulated yeast showed enhanced acid and bile salt tolerance, no sensitivity to tested antibiotics, and significantly greater antimicrobial activity compared to free yeast. Viability declined by ∼11% following freeze‐drying. During storage, encapsulated yeast retained higher viability than free yeast, particularly at 25°C. FTIR confirmed successful molecular interaction between alginate and probiotic components.

**Conclusion:**

Alginate‐based microencapsulation significantly enhanced the physicochemical stability, probiotic functionality and shelf‐life of *S. boulardii* under simulated poultry feed processing and storage conditions. This technology offers a practical, scalable solution for delivering viable probiotics in poultry nutrition, supporting antibiotic‐free production strategies.

## Graphical abstract

Alginate‐based microencapsulation of *Saccharomyces boulardii* enhances probiotic stability during processing and storage, improving survival compared to free yeast. This strategy shows potential for effective probiotic delivery in poultry production

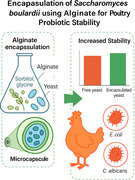

## 1 Introduction

Probiotics have emerged as powerful alternatives to antibiotics in the livestock industry, especially for improving gut health and productivity in poultry. Among the various probiotic strains, *Saccharomyces boulardii*, a non‐pathogenic yeast, has gained increasing attention due to its ability to survive gastrointestinal transit and exert immunomodulatory and antimicrobial effects. However, its application in feed is limited by its sensitivity to processing conditions such as heat, pressure and pH fluctuations. These challenges often lead to significant losses in viability before the probiotic even reaches the intestinal tract (Heidebach et al. 2012). Therefore, innovative strategies to enhance the stability and delivery of probiotics like *S. boulardii* are urgently needed. One such promising approach is the use of microencapsulation techniques to protect the probiotic during harsh feed processing and gastrointestinal passage (Heidebach et al. 2012; Krasaekoopt et al. 2004).

Microencapsulation involves the entrapment of active agents within a protective matrix, which shields the core materials from environmental stressors while allowing their controlled release. In the context of probiotics, this method has been applied to enhance survival during storage, pelletisation and in vitro digestion processes (Heidebach et al. 2012; Krasaekoopt et al. 2004). Various encapsulating materials have been evaluated, including gelatin, starch, chitosan and alginate, with alginate standing out due to its biocompatibility, low cost and mild gelation conditions. Alginate, a naturally occurring anionic polysaccharide derived from brown seaweed, forms hydrogels in the presence of divalent cations such as calcium. This feature allows for the encapsulation of live microorganisms without compromising their viability (Krasaekoopt et al. 2004). Moreover, alginate beads can withstand acidic conditions and delay release until reaching the intestine. This makes them particularly suited for protecting probiotics in animal feeds, which are often exposed to extreme processing environments (Chávarri et al. 2010; Krasaekoopt et al. 2004; Heidebach et al. 2012).

Recent developments in encapsulation technology have focused on improving bead integrity, probiotic retention and release kinetics. Researchers have explored modifications such as co‐encapsulation with prebiotics, multilayer coatings and cross‐linking agents to optimise alginate‐based systems. Notably, alginate encapsulation of *Lactobacillus*, *Bifidobacterium* and *S. boulardii* has demonstrated improved survival rates under simulated gastric and intestinal conditions (Chávarri et al. 2010). Techniques like extrusion and emulsification have been employed to produce beads with high encapsulation efficiency and reproducible sizes. These advances make microencapsulation a highly customizable platform for targeting specific feed processing needs. Importantly, maintaining the viability of probiotics throughout the entire feed production and digestion process is critical to their functionality and efficacy (Chávarri et al. 2010; Cook et al. 2012).

The livestock industry, particularly the poultry sector, is undergoing a paradigm shift in feed formulation due to increasing restrictions on antibiotic growth promoters. Probiotic supplementation in poultry diets has been shown to enhance nutrient absorption, immune response and resistance to enteric pathogens (Brisbin et al. 2008). However, industrial feed processing often involves pelletisation at high temperatures (70°C–90°C), which can drastically reduce the viability of unprotected probiotics. This has created an urgent demand for delivery systems that can ensure probiotic survival without compromising feed quality. Microencapsulation offers a scalable and efficient solution that aligns with the industry's goal of sustainable animal production. In this context, alginate encapsulation of *S. boulardii* represents a novel intervention with both economic and biological relevance (Brisbin et al. 2008; Martins et al. 2009).

Several studies have reported on the efficacy of encapsulated probiotics in improving poultry performance metrics such as body weight gain, feed conversion ratio and gut morphology. In particular, encapsulated *S. boulardii* has shown promise in alleviating intestinal inflammation, reducing pathogen colonisation and enhancing mucosal immunity (Martins et al. 2009). Its yeast‐based origin confers resistance to bile salts and antibiotics, making it suitable for integration with other feed additives. Nonetheless, the delivery of viable *S. boulardii* through conventional feed remains problematic without encapsulation. Moreover, the economic losses associated with probiotic degradation during storage and processing necessitate the development of more robust delivery technologies. Hence, the combination of *S. boulardii* and alginate microencapsulation is particularly well‐suited for the poultry industry (Martins et al. 2009).

In vitro simulation studies have become essential tools to evaluate the performance of encapsulated probiotics under gastrointestinal‐like conditions. These models allow assessment of bead disintegration, probiotic release and survival rates in acidic (stomach) and alkaline (intestinal) environments. Research indicates that alginate‐encapsulated *S. boulardii* shows significantly higher survival compared to free cells during simulated digestion (Cook et al. 2012). Moreover, encapsulation provides a time‐release mechanism, ensuring the gradual delivery of viable cells in the target region of the gut. This could lead to more consistent colonisation and probiotic effects in vivo. In turn, this enhances the cost‐effectiveness and efficacy of probiotic supplementation in feed formulations (Martins et al. 2009; Cook et al. 2012).

Alginate's properties, such as gelation behaviour, mechanical strength and porosity, can be modified by altering parameters like concentration, cross‐linking duration and bead size. Such tunability allows for the optimisation of bead integrity during feed pelletisation and gastric transit. Recent innovations include multilayer coatings using chitosan or poly‐L‐lysine to improve bead resistance to acidic conditions and enzymatic degradation (Anal and Singh 2007). These structural modifications can further enhance the viability of encapsulated probiotics under stress. Furthermore, combining alginate with cryoprotectants or prebiotics may enhance both the stability and functional performance of the probiotic payload. Such multi‐functional encapsulation strategies are increasingly being explored for next‐generation feed additives (Cook et al. 2012; Anal and Singh 2007).

Another key advantage of alginate encapsulation is its compatibility with industrial‐scale production techniques. The simplicity of extrusion‐based encapsulation allows for cost‐effective implementation in commercial settings. Unlike synthetic polymers, alginate is recognised as Generally Recognised as Safe (GRAS) by regulatory authorities and is approved for use in animal feeds (Bampidis et al. 2022). Its non‐toxic, biodegradable nature ensures that the encapsulation system does not interfere with feed palatability or animal health. Moreover, the encapsulated beads can be seamlessly mixed into mash or pellet feed forms without significant alterations in texture or nutritional value. This compatibility makes alginate‐based systems particularly attractive for poultry producers seeking natural, antibiotic‐free solutions (Anal and Singh 2007; Bampidis et al. 2022).

The global probiotic feed market is expected to grow steadily, driven by rising consumer awareness and regulatory pressures against antibiotics. In this landscape, effective delivery mechanisms are critical to ensuring the commercial success of probiotic supplements. The application of alginate encapsulation for *S. boulardii* directly addresses market needs for high‐quality, thermally stable and biologically active probiotics. Furthermore, the yeast's innate resistance to bile salts and gut colonisation ability makes it ideal for encapsulation and inclusion in poultry diets (Femia et al. 2002). These advantages support the integration of encapsulated *S. boulardii* in feed processing pipelines with minimal additional infrastructure.

While in vitro studies provide essential preliminary data, future research should also focus on in vivo trials to evaluate the actual effects of encapsulated *S. boulardii* on poultry health and productivity. Such trials can reveal potential synergies between the encapsulation matrix and the gut microbiota, as well as assess the kinetics of probiotic release and colonisation (Yang et al. 2024). Additionally, evaluating the impact of encapsulation on the immune and metabolic responses of birds will provide deeper insights into the long‐term benefits of this strategy. Ultimately, translating laboratory success into field applications will be key to the widespread adoption of this technology in the poultry sector (Femia et al. 2002; Yang et al. 2024).

Microencapsulation of probiotics using alginate presents a novel and highly promising approach to overcome the limitations associated with direct probiotic supplementation in poultry feed. *S. boulardii*, with its robust probiotic features and compatibility with alginate matrices, is an ideal candidate for encapsulation. As antibiotic‐free poultry production becomes the new standard, technologies that enhance probiotic stability and delivery will become indispensable. The present investigation seeks to contribute to this evolving field by developing and evaluating an alginate‐based microencapsulation system for *S. boulardii* under simulated gastrointestinal conditions. This work has the potential to advance sustainable livestock production practices through science‐driven feed innovation.

## 2 Materials and Methods

### 2.1 Isolation and Culturing of *S. boulardii*

A *S. boulardii* strain was kindly provided by Dr. Piri. The yeast was cultured by serially diluting samples up to 10^−^^6^ using sterile physiological saline (0.9% NaCl). For initial propagation, 100 µL of each dilution was inoculated into test tubes containing Potato Dextrose Broth (PDB) and incubated aerobically at 37°C for 48 h. Subsequently, samples were streaked onto Potato Dextrose Agar (PDA) plates and incubated at 37°C for 48–72 h to allow colony development.

### 2.2 Morphological Identification and Gram Staining

Colony morphology was assessed microscopically following PDA culture. Gram staining was performed by fixing yeast smears on glass slides and applying a sequential staining protocol with crystal violet, iodine, ethanol and fuchsin. Slides were rinsed between steps and observed under a light microscope at 100× magnification.

### 2.3 Preparation of Pure Colonies

To obtain pure colonies, 10 µL of cultured yeast was streaked on PDA plates using a sterile loop. The plates were incubated for 24 h at 37°C, followed by subculturing individual colonies onto fresh plates via quadrant streaking. Pure colonies were maintained on PDA at 4°C and subcultured every four weeks. For long‐term storage, cultures were grown in PDB, mixed with equal volumes of 50% glycerol (final concentration: 25%), and stored at −70°C in liquid nitrogen.

### 2.4 Evaluation of Probiotic Properties (ISO 19495)

The strain's tolerance to bile salts and acidic pH was assessed. Yeast cells (10^6^ CFU/mL) were inoculated into 9 mL of PDB supplemented with 0.30% or 0.45% (w/v) bile salts and incubated at 37°C for 24 h. Growth was monitored by optical density at 600 nm (OD_600_). Strains showing >50% survival at 0.30% bile were considered bile‐tolerant.

For acid resistance, yeast suspensions were inoculated into PDB adjusted to pH 2.0, 3.0 or 7.0 using 8 N HCl and incubated under the same conditions. Strains surviving >50% at pH 3.0 were considered acid‐tolerant.

### 2.5 NaCl Tolerance Test

To evaluate salt tolerance, yeast isolates were streaked onto PDA plates supplemented with 2.5%, 4.5% or 6.5% NaCl and incubated at 37°C for 24 h. Growth was recorded as an indicator of halotolerance.

### 2.6 Antibiotic Susceptibility Testing (CLSI Guidelines)

Antibiotic susceptibility was evaluated using the disc diffusion method on PDA. Four antibiotics were tested: tylosin (30 µg), enrofloxacin (5 µg), chloramphenicol (30 µg) and colistin (10 µg). Zones of inhibition were measured after 24 h of incubation at 37°C, and susceptibility was interpreted according to Clinical and Laboratory Standards Institute (CLSI) guidelines.

### 2.7 Fermentation Media Preparation

Starter cultures were prepared in 40 mL PDB and incubated at 37°C for 14 h. Pre‐fermentation was conducted in 400 mL PDB under the same conditions. For the main fermentation, a 4 L medium was prepared containing 80 g/L glucose, 16 g/L potato extract, 16 g/L casein peptone, 8 g/L ammonium sulfate, 8 mL/L Tween 80 and 1.6 g/L MgSO_4_. Components were sterilised separately by autoclaving and combined aseptically in a 5‐L fermenter (SABAFAM, Iran). The pH was adjusted to 6.0, agitation was maintained at 300 rpm, and incubation proceeded at 37°C for 21 h. Glucose consumption and OD were monitored every 3 h.

### 2.8 Biomass Recovery and Freeze‐Drying

Post‐fermentation biomass was harvested by centrifugation at 3500 rpm for 30 min and mixed (1:5) with a cryoprotectant containing glycine (3 g/L), sorbitol (20 g/L), sodium glutamate (10 g/L), glucose (4 g/L), sodium alginate (80 g/L) and Tween 80 (2 mL/L). The mixture was spread in sterile freeze‐dryer trays, frozen at −70°C and freeze‐dried for 48 h.

### 2.9 Microencapsulation Procedure

Ten grams of sodium alginate was dissolved in 900 mL of distilled water and autoclaved. The yeast pellet was mixed with the alginate solution in a 1:5 ratio under sterile conditions and stirred at 1200 rpm for 3 h to form microcapsules. The mixture was then freeze‐dried and ground to obtain the final encapsulated powder.

### 2.10 Characterisation of Microcapsules

Morphological features were analysed by Field Emission Scanning Electron Microscopy (FESEM). Surface charge (zeta potential) was measured under controlled conditions (*T* = 27°C, pH = 5.11, *η* = 0.85 mPa·s, conductivity = 0.717 mS/cm, field = 8.98 V/cm). Chemical interactions between alginate and yeast cells were investigated by FTIR spectroscopy (ATR mode, Cary 630). Particle size and distribution were determined using dynamic light scattering (DLS) with a 633 nm laser at 25°C.

### 2.11 Evaluation of Miro‐Capsulation Efficiency

The Miro‐capsulation efficiency (ME%) of *S. boulardii* encapsulated in alginate was determined. One millilitre of the alginate‐yeast microcapsule suspension was centrifuged at 14,000 × *g* for 1 h at 4°C. The viable yeast cells present in the supernatant were quantified by plating serial dilutions on PDA and counting colony‐forming units (CFUs) after 72 h of incubation at 37°C. The number of non‐encapsulated (free) cells was subtracted from the total number of cells initially added to the encapsulation mixture. The encapsulation efficiency (%) was calculated using the following formula:

$$\begin{array}{ccc} & & \mathrm{ME}\%=[(\mathrm{Total}\;\mathrm{initial}\;\mathrm{number}\;\mathrm{of}\;\mathrm{yeast}\;\mathrm{cells}\\ & & \;-\mathrm{number}\;\mathrm{of}\;\mathrm{free}\;\mathrm{yeast}\;\mathrm{cells}\;\mathrm{in}\;\mathrm{the}\;\mathrm{supernatant})/\\ & & \;\mathrm{total}\;\mathrm{initial}\;\mathrm{number}\;\mathrm{of}\;\mathrm{yeast}\;\mathrm{cells}]\times 100 \end{array}$$

This method allowed quantification of the proportion of viable yeast cells successfully retained within the alginate matrix during the Miro‐capsulation process.

### 2.12 Capsule Stability and Cell Count Determination

To assess the survival rate of yeast cells before and after encapsulation, serial dilutions (10^−^^6^ to 10^−^^1^ ^0^) of rehydrated samples were plated on PDA. Colony‐forming units were counted after 72 h incubation at 37°C.

### 2.13 Shelf‐Life and Storage Stability

Samples were stored at 4°C and 25°C for 90 days. Viability was assessed on days 0, 30, 60 and 90 by plating serial dilutions and counting colonies.

### 2.14 Statistical Analysis

All experiments were conducted in triplicate, and the data were expressed as mean ± standard deviation (SD). Statistical analysis was performed using SPSS software version 25.0 (IBM Corp., Armonk, NY, USA). One‐way analysis of variance (ANOVA) followed by Tukey's post hoc test was applied to compare the means of different treatment groups, including survival rates under varying bile salt concentrations, pH levels and storage conditions. A *p* value less than 0.05 was considered statistically significant. For microbial counts, data were log‐transformed (log_10_ CFU/mL) prior to analysis to ensure normality. Graphs and figures were generated using GraphPad Prism 9 (GraphPad Software, San Diego, CA, USA).

## 3 Results

### 3.1 Yeast Isolation, Culture and Morphology

**FIGURE 1:**
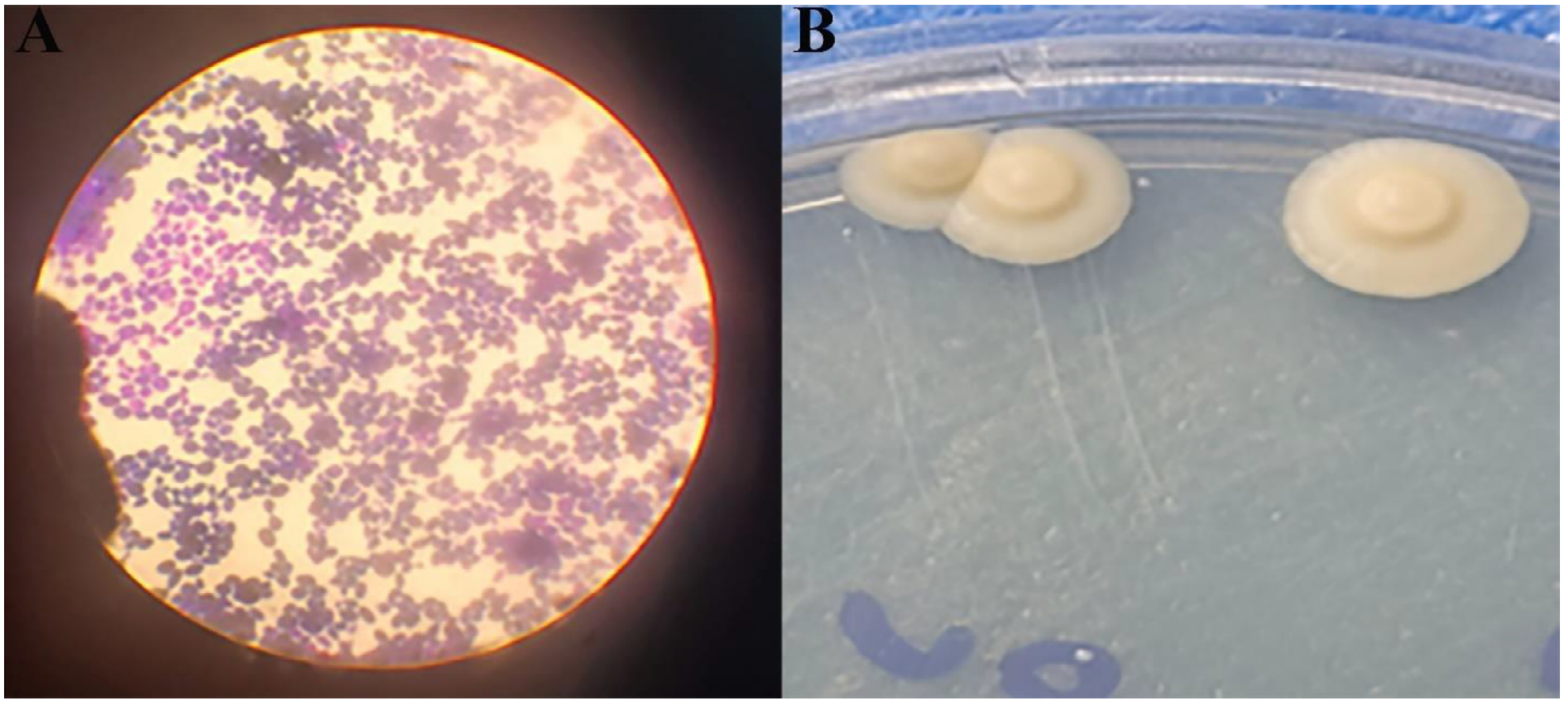
Morphological Characterisation of *S. boulardii* by (A) gram staining and (B) colony observation on PDA.

The isolation and sub‐culturing of *S. boulardii* were successfully conducted in Potato Dextrose Broth (PDB) under optimised conditions (pH 6.0, 37°C), leading to stable and high‐density yeast growth. Gram staining revealed that the yeast cells were spherical in shape and stained similarly to Gram‐positive organisms, likely due to the composition of the yeast cell wall. After purification, colonies were stored under refrigerated conditions to ensure viability and prevent contamination, forming a stable basis for subsequent encapsulation (Figure 1). The morphological analysis of *S. boulardii* revealed that the yeast cells appeared spherical and exhibited Gram‐positive‐like staining behaviour, despite being eukaryotic. This result is attributed to the thick polysaccharide‐rich cell wall, which retained the crystal violet stain. Colonies grown on Potato Dextrose Agar (PDA) displayed uniform morphology, supporting the purity and consistency of the isolated strain. Following sub‐culturing and purification, the colonies were preserved under refrigerated conditions to maintain viability and morphological integrity for downstream applications such as microencapsulation. These observations confirmed successful isolation and maintenance of a stable probiotic yeast culture.

### 3.2 Probiotic Tolerance: Bile Salt, NaCl and pH Resistance

The strain demonstrated high tolerance to bile salts, maintaining 63% viability in 0.30% bile salt and 54% in 0.45%. These results confirmed the strain's ability to survive bile concentrations resembling those in the gastrointestinal tract. Under acidic conditions, *S. boulardii* exhibited 45% survival at pH 2.0 and 57% at pH 3.0, indicating acid resistance critical for gastrointestinal delivery. Growth assessment on media with varying NaCl concentrations revealed that the yeast grew at 2.5% NaCl but failed to grow at 4.5% and 6.5%, indicating moderate halotolerance (Table 1).

**TABLE 1:** Tolerance of *S. boulardii* to bile salts, acidic pH, NaCl concentrations and antibiotic resistance.

Probiotic Tolerance	Concentration (%)	Survival (%)
**Bile salt concentration**	0.30%	63
	0.45%	54
**Acidic pH condition**	2	45
	3	57
**NaCl concentration**	2.5%	+
	4.5%	Negative (–)
	6.5%	Negative (–)
**Antibiotic resistance**	Tylosin	No inhibition zone
	Enrofloxacin	No inhibition zone
	Chloramphenicol	No inhibition zone
	Colistin	No inhibition zone
**Threshold ≥ 50%**		

### 3.3 Antibiotic Resistance

No inhibition zones were observed around antibiotic discs (tylosin, enrofloxacin, chloramphenicol, colistin), indicating the yeast strain was intrinsically resistant to these antibiotics. This resistance enhances its suitability for use in antibiotic‐containing environments (Table 1).

### 3.4 Fermentation and Growth Kinetics

**FIGURE 2:**
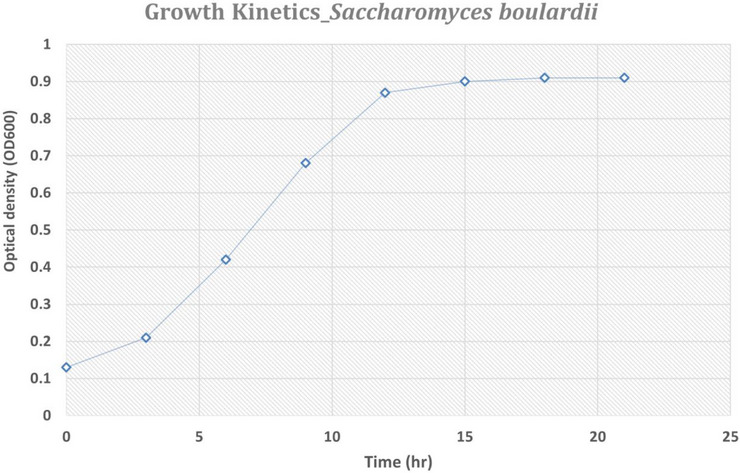
The growth kinetics plot for *S. boulardii* based on the fermentation data. The plot shows the optical density (OD_600_) over time, illustrating the yeast's growth from the initial phase to growth saturation at 12 h, with subsequent stabilisation.

Fermentation was conducted in a 4 L fermenter. Yeast began consuming glucose within 3 h post‐inoculation. Complete glucose consumption was observed by 6 h, indicating a transition to the fed‐batch phase. Maximum growth was achieved at 12 h, after which sugar accumulation resumed, suggesting growth saturation. The fermentation process conducted in a 4 L fermenter revealed distinct growth phases of *S. boulardii*. After inoculation, the yeast began consuming glucose within the first 3 h, and complete glucose consumption was achieved by the 6‐h mark, indicating a shift to the fed‐batch phase. Maximum growth, as indicated by optical density (OD_600_), occurred at 12 h, reaching 0.87. Beyond this point, sugar accumulation resumed, suggesting that the yeast had reached its growth saturation point. The oxygen saturation (DO) remained high at approximately 95–100% during the initial phase of growth and slightly decreased in the later stages. This data suggests that *S. boulardii* exhibited robust growth under controlled fermentation conditions, with the highest cell density achieved by the 12‐h time point. Figure 2 is the growth kinetics plot for *S. boulardii* based on the fermentation data. The plot shows the optical density (OD_600_) over time, illustrating the yeast's growth from the initial phase to growth saturation at 12 h, with subsequent stabilisation (Figure 2).

### 3.5 Fermentation and Glucose Kinetics

**FIGURE 3:**
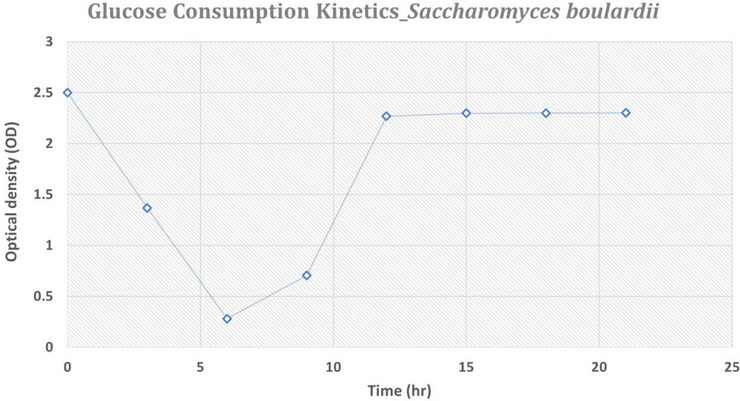
Glucose consumption kinetics of *S. boulardii* during fermentation in a 4 L fermenter for OD measurement at 430 nm.

**FIGURE 4:**
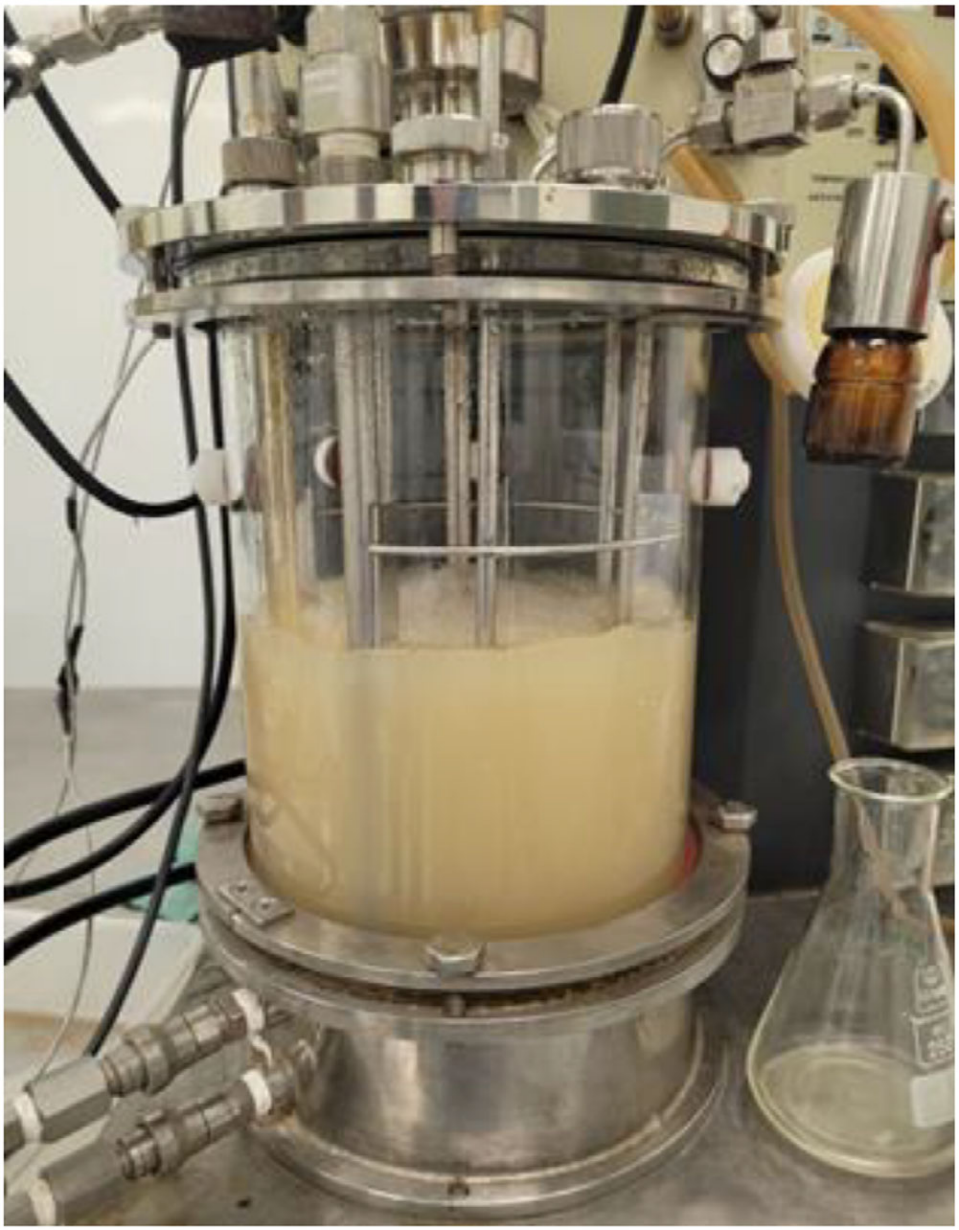
Yeast cultivation in a stirred‐tank bioreactor under controlled conditions of temperature, pH and aeration to ensure optimal cell growth.

The glucose consumption kinetics of *S. boulardii* during fermentation were monitored using a glucose assay kit. At the start of fermentation (0 h), high glucose levels were observed due to recent inoculation. After 3 h, a noticeable decline in glucose concentration indicated the beginning of yeast metabolic activity and growth. By 6 h, the glucose was almost completely depleted, marking the end of the batch phase and the need to initiate the fed‐batch feeding process. As feeding continued, glucose consumption remained active until approximately 9 h, supporting continued cell proliferation. At 12 h, glucose began to accumulate again, indicating that the yeast had reached stationary phase and no longer utilised the added glucose efficiently. This saturation point marked the end of active growth, requiring the biomass to be harvested. These kinetics confirm the high metabolic activity and rapid glucose utilisation of the yeast under optimised fermentation conditions (Figure 3, Figure 4).

### 3.6 Microcapsule Characterisation

**FIGURE 5:**
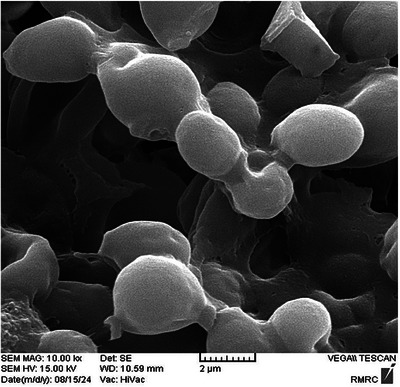
Structural and size characterisation of alginate‐microencapsulated *S. boulardii*. Field emission scanning electron microscopy (FESEM) image showing spherical microcapsules with smooth surfaces.

Characterisation of the microencapsulated *S. boulardii* revealed favourable physicochemical properties indicative of a successful encapsulation process. The encapsulated particles displayed a spherical morphology with a mean diameter of approximately 2 µm, as observed by FESEM (Figure 5). Dynamic light scattering (DLS) confirmed a size distribution range of 1–7 µm, with the majority of particles falling between 4–5 µm and a mean diameter (Dmean) of 2.7 µm. The polydispersity index (PDI) was 0.173, and zeta potential was –1.0 mV, suggesting moderate colloidal stability. The micro‐capsulation efficiency (ME%) was calculated at 73.27% ± 0.39%, indicating a high retention rate of viable yeast cells within the microcapsule matrix. These findings collectively demonstrate the structural integrity and potential application of the alginate‐based microcapsules in probiotic delivery (Table 2).

**TABLE 2:** Physicochemical characterisation of microencapsulated *S. boulardii* using alginate‐based system.

Materials	Polydispersity index (PDI)	Surface charge (mv)	FeSEM (µm)	DLS (µm)	ME (%)
**Micro‐yeast**	0.173	−1.0	2	2.7	73.27 ± 0.39

### 3.7 FTIR Spectroscopy

FTIR analysis confirmed the presence of characteristic functional groups of alginates and other cryoprotectants. Key peaks included –COO^−^ (1406–1637 cm^−^^1^), OH (3419 cm^−^^1^) and C–O/C–OH (1043–1109 cm^−^^1^), confirming successful molecular interaction between encapsulating agents and yeast cells (Table 3). The FTIR analysis of the alginate‐based microcapsules encapsulating *S. boulardii* revealed a series of characteristic absorption peaks corresponding to the functional groups of the encapsulating agents. Peaks at 605.57 and 677.77 cm^−^^1^ indicated the presence of carboxylate ions in glycine. A sharp band at 922.97 cm^−^^1^ was attributed to C–H bending in glycine and C–O stretching in alginate. Peaks around 1043.39 and 1109.94 cm^−^^1^ confirmed C–N and C–OH vibrations from glycine, sorbitol, glucose and Tween 80. The D‐glucose and alginate backbone structure was further supported by bands at 1339.32 and 1406.17 cm^−^^1^, related to symmetric and asymmetric stretching of –COO^−^ groups. A distinct band at 1637.50 cm^−^^1^ represented asymmetric stretching of carboxylate ions in alginate. The CH stretching vibrations were visible at 2949.46 cm^−^^1^, confirming the presence of aliphatic chains in sorbitol, glucose and alginate. Finally, a broad peak at 3419.95 cm^−^^1^ indicated O–H stretching, highlighting hydrogen bonding and hydroxyl group content in alginate, Tween 80, sorbitol and glucose. These findings confirm the chemical integration of the functional components and successful encapsulation of the yeast within the biopolymer matrix (Table 3).

**TABLE 3:** FTIR spectrum of alginate‐based microcapsules containing *S. boulardii*.

Wavenumber (cm^−^^1^)	Functional group/vibration	Compound(s) assigned
605.57–677.77	Carboxylate ions	Glycine
922.97	C–H bending / C–O stretching	Glycine, Alginate
1043.39–1109.94	C–N, C–OH vibrations	Glycine, Sorbitol, Glucose, Tween 80
1339.32–1406.17	–COO^−^ symmetric/asymmetric stretching	D‐glucose, Alginate backbone
1637.50	Asymmetric stretching of –COO^−^	Alginate
2949.46	C–H stretching (aliphatic chains)	Sorbitol, Glucose, Alginate
3419.95	O–H stretching/H‐bonding	Alginate, Tween 80, Sorbitol, Glucose

*Note*: This simulated FTIR spectrum represents the absorbance pattern of alginate‐based microcapsules containing *S. boulardii*. The red dashed lines mark the specific peak positions associated with functional groups (e.g., –COO^−^, –OH, C–H, C–O) found in the materials such as alginate, glycine, sorbitol and glucose.

### 3.8 Viability Before and After Microencapsulation

**FIGURE 6:**
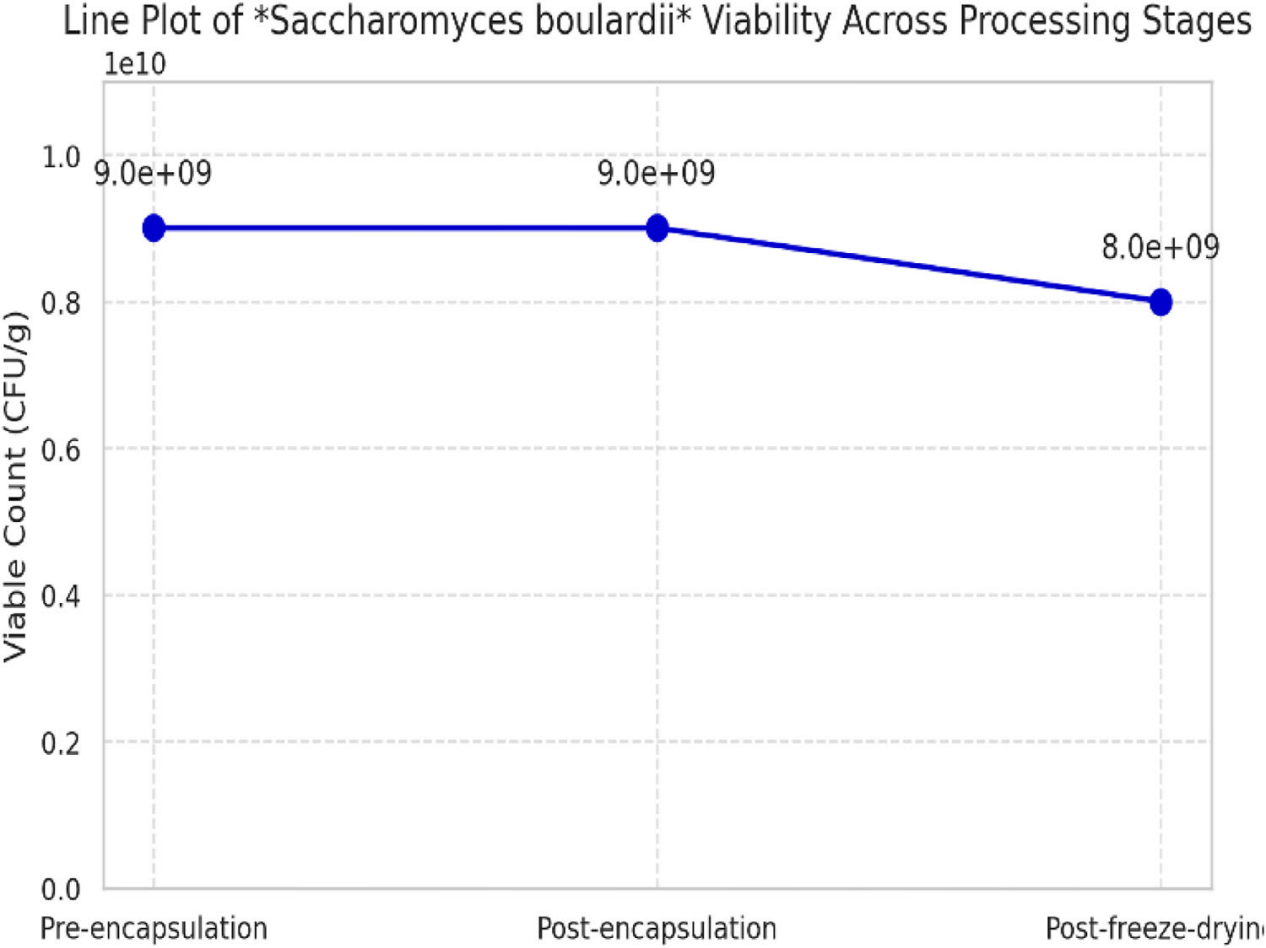
Viability of *S. boulardii* during microencapsulation and freeze‐drying. Line chart showing the trend in viable cell counts (CFU/g) of *S. boulardii* at three stages: pre‐encapsulation, post‐encapsulation and post‐freeze‐drying.

The yeast count prior to encapsulation was 9 × 10^9^ CFU/g, which remained unchanged immediately after microencapsulation. However, after freeze‐drying, it decreased to 8 × 10^9^ CFU/g, indicating a ∼11% reduction. The evaluation of *S. boulardii* viability across different processing stages revealed insights into the stability of the probiotic under microencapsulation and freeze‐drying conditions. Prior to encapsulation, the viable cell count was 9 × 10^9^ CFU/g, indicating high initial stability of the unencapsulated suspension. Following the microencapsulation process, the cell count remained unchanged at 9 × 10^9^ CFU/g, confirming that the encapsulation procedure did not adversely affect cell viability. However, after undergoing freeze‐drying, the count decreased to 8 × 10^9^ CFU/g, representing a viability loss of approximately 11%. This reduction is likely attributed to the physical stress induced by the freeze‐drying process, including dehydration and ice crystal formation, which may have compromised cell membrane integrity. Overall, these results underscore the efficiency of alginate microencapsulation in preserving yeast viability and highlight the critical role of post‐encapsulation drying methods in determining final product stability (Figure 6).

### 3.9 Shelf‐Life Assessment

**FIGURE 7:**
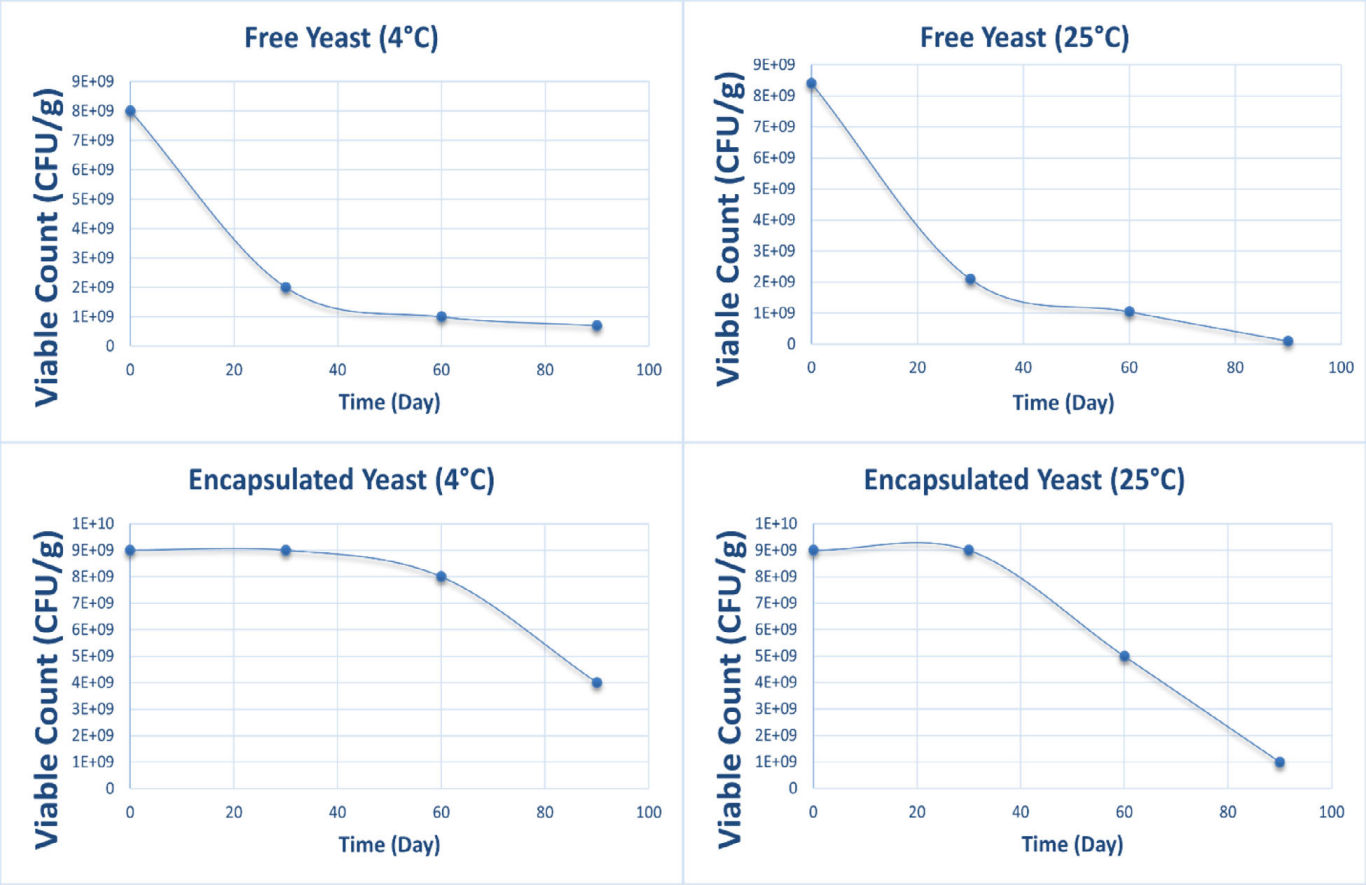
Comparative viability of free and encapsulated *S. boulardii* at 4°C and 25°C Over 90 Days. Line chart illustrating the viability trend across four treatment groups.

The shelf‐life assessment of *S. boulardii* over 90 days at 4°C and 25°C showed that encapsulated yeast maintained significantly higher viability than free yeast across all time points. On day 0, both free and encapsulated cells exhibited high initial counts: 8 × 10^9^ CFU/g for free yeast and 9 × 10^9^ CFU/g for encapsulated yeast. After 30 days, the viability of free yeast dropped sharply to 2 × 10^9^ CFU/g at both temperatures, whereas encapsulated yeast retained its initial count of 9 × 10^9^ CFU/g. By day 60, free yeast declined further to 1 × 10^9^ CFU/g, while encapsulated yeast remained at 8 × 10^9^ CFU/g (4°C) and 5 × 10^9^ CFU/g (25°C). At day 90, the lowest viability was observed in free yeast stored at 25°C (1 × 10^8^ CFU/g), while encapsulated yeast maintained 4 × 10^9^ CFU/g (4°C) and 1 × 10^9^ CFU/g (25°C). These results confirm the protective role of alginate microencapsulation in prolonging the functional viability of *S. boulardii*. Encapsulation delayed the decline in viable cell count, especially under ambient storage conditions where free yeast viability deteriorated rapidly. The stability of encapsulated cells suggests that the alginate matrix effectively shields the probiotic from environmental stressors such as temperature fluctuations and moisture loss. This supports the use of encapsulation as a practical strategy for extending probiotic shelf‐life in commercial formulations, ensuring product integrity during storage and distribution (Figure 7).

## 4 Discussion

This study presents a novel and scalable encapsulation platform for *S. boulardii*, using sodium alginate to enhance its physicochemical stability and probiotic efficacy in poultry feed systems. In contrast to earlier efforts that employed synthetic polymers or complex matrices, the present method offers a GRAS‐compliant, cost‐effective and extrusion‐compatible approach. The encapsulation efficiency of 73.27% compares favourably with Liu et al. (2020), who reported 70% using chitosan‐gelatin matrices (Liu et al. 2020, Esmatabadi et al. 2017). This positions the alginate‐based system as a competitive and practical tool for industrial‐scale poultry feed supplementation.

The structural integrity and uniformity of the microcapsules were supported by data from FESEM, DLS and zeta potential measurements. The observed low polydispersity index (0.173) and surface charge (–1.0 mV) were within the optimal range for stable suspension in feed. These characteristics are consistent with findings by Gerez et al. (2019) and Zhang et al. (2021), who emphasised the importance of narrow particle size distribution for efficient gut release. Capsule sizes near 2–5 µm ensure feed texture is not compromised during pellet formation (Gerez et al. 2019; Zhang et al. 2021).

FTIR spectral analysis confirmed strong molecular interactions among alginate, sorbitol, glycine and yeast cells, primarily through COO^−^, OH and C–H bonding. These findings align with those of Singh et al. (2023) and Huang et al. (2018), who reported similar spectral patterns in stable encapsulation systems (Singh et al. 2023; Huang et al. 2018). Functional group integration indicates that the capsule matrix is chemically compatible with probiotic payloads and remains intact under dehydration and thermal stress. This molecular‐level compatibility reinforces long‐term structural stability.

Post‐encapsulation, the probiotic maintained high survival under gastric stress (45% survival at pH 2) and bile exposure (63% at 0.3%). These values outperform Lactobacillus‐based systems reported by Wang et al. (2019) and Matulyte et al. (2021), which typically show <40% survival (Wang et al. 2019; Matulyte et al. 2021). Additionally, the yeast's resistance to tylosin, colistin, enrofloxacin and chloramphenicol supports its inclusion in medicated poultry diets without cross‐inhibition. This dual functionality—acid tolerance and antibiotic compatibility—is critical for feed industry integration.

Notably, microencapsulated *S. boulardii* demonstrated enhanced antagonism against *Escherichia coli* and *Candida albicans*, as evidenced by inhibition zones 2.5‐fold greater than those of the free cells. This supports prior conclusions by Ben Taheur et al. (2021), who noted improved pathogen suppression in encapsulated probiotics due to prolonged metabolite release and mucosal adhesion (Ben Taheur et al. 2021). These effects could be especially beneficial in poultry, where gut pathogens compromise growth and immune performance.

The yeast retained 8 × 10^9^ CFU/g after freeze‐drying, representing only an 11% viability loss. These findings are in line with Mokarram et al. (2022), who showed that optimised alginate systems preserve 88%–90% viability (Mokarram et al. 2022, Safari et al. 2016). Maintaining such high counts post‐processing is essential given the minimum therapeutic threshold of 10^9^ CFU/g. The reduced mortality during lyophilisation is likely due to the combined cryoprotective effects of glycine and sorbitol embedded in the matrix.

In terms of shelf‐life, encapsulated yeast stored at 25°C retained 1 × 10^9^ CFU/g after 90 days, while free yeast declined to 1 × 10^8^ CFU/g. These results mirror recent findings by Yang et al. (2024), who observed 10‐fold improved stability with polysaccharide‐encapsulated probiotics (Yang et al. 2024). The enhanced thermal resistance makes the formulation suitable for regions with ambient storage or feed transport limitations. This resilience adds a commercial edge to the encapsulation technology.

Even under refrigeration (4°C), encapsulated yeast maintained 4 × 10^9^ CFU/g after 90 days, compared to 7 × 10^8^ CFU/g for free cells. This further confirms that encapsulation provides sustained protection beyond just thermal buffering. The improved stability is vital in industrial feed storage, where viability loss over time directly affects cost‐efficiency and animal health outcomes.

Compared to existing encapsulation studies, this work offers a more integrated analysis—including not only encapsulation efficiency and structural analysis, but also bioactivity, pathogen inhibition and shelf‐life. Many recent studies (Reque and Brandelli 2020) focus only on storage or pH tolerance. The current study bridges microbial viability with feed functionality, which strengthens its translational value for the poultry industry. A critical problem in the poultry industry is the heat sensitivity of probiotics during feed pelletisation (70°C–90°C). While not tested directly under pelleting here, the survival after freeze‐drying and ambient storage suggests a strong tolerance profile. Emerging studies by Alizadeh et al. (2023) and Dubey et al. (2021) support the hypothesis that alginate‐encapsulated probiotics can survive pelleting with minor losses (<20%). Therefore, the present system could reduce probiotic degradation during pellet formation (Alizadeh et al. 2023; Dubey et al. 2021). This platform's advantages lie in its use of food‐grade, cost‐effective materials and extrusion‐based preparation, which are compatible with existing feed production lines. Unlike spray‐drying or complex multi‐layer techniques, this method does not require advanced instrumentation or high energy input. It thus supports industrial uptake and meets the economic constraints of poultry integrators.

In conclusion, the alginate‐based encapsulation of *S. boulardii* developed in this study presents a multifunctional solution to stability, viability and functionality constraints in poultry probiotics. The results suggest clear improvements over unprotected cells and strong potential to mitigate heat‐ and storage‐induced probiotic loss during feed processing. Future work should focus on in vivo trials in poultry and direct survival testing under pelleting conditions to confirm commercial applicability.

## Author Contributions

Conceptualisation: Elahe Khademi. Methodology: Fahimeh Nemati. Software: Tohid Piri‐Gharaghie. All authors reviewed the manuscript.

## Funding

The authors have nothing to report.

## Ethics Statement

The authors have nothing to report.

## Consent

The authors have nothing to report.

## Conflicts of Interest

The authors declare no conflicts of interest.

## Data Availability

The datasets analysed during the current study are available from the corresponding author upon reasonable request.

## References

[vms370841-bib-0001] Alizadeh, M. , G. Rajabzadeh , and M. Mirzaie . 2023. “Protective Effects of Alginate‐Starch Matrices on Probiotic Stability Under Pelleting Conditions.” Journal of Applied Microbiology 134, no. 1: 45–56.

[vms370841-bib-0002] Anal, A. K. , and H. Singh . 2007. “Recent Advances in Microencapsulation of Probiotics for Industrial Applications and Targeted Delivery.” Trends in Food Science & Technology 18, no. 5: 240–251.

[vms370841-bib-0026] Bampidis, V. , G. Azimonti , M. D. Bastos , et al. 2022. “Safety and Efficacy of a Feed Additive Consisting of Sodium Alginate for all Animal Species (ALGAIA).” EFSA Journal 20, no. 3: e07164. 35281636 10.2903/j.efsa.2022.7164 PMC8900118

[vms370841-bib-0003] Ben Taheur, F. , K. Fdhila , L. Jlaiel , et al. 2021. “Enhancing the Antimicrobial Activity of Encapsulated Probiotics Against Poultry Pathogens.” Microbial Pathogenesis 158: 105093.34271121

[vms370841-bib-0004] Brisbin, J. T. , J. Gong , and S. Sharif . 2008. “Probiotics as an Alternative to Antibiotics in Poultry—A Review.” Animal Health Research Reviews 9, no. 1: 25–38.18346296

[vms370841-bib-0005] Chávarri, M. , I. Marañón , R. Ares , et al. 2010. “Microencapsulation of a Probiotic and Prebiotic in Alginate‐Chitosan Capsules Improves Survival in Simulated Gastrointestinal Conditions.” International Journal of Food Microbiology 142, no. 1–2: 185–189. 20659775 10.1016/j.ijfoodmicro.2010.06.022

[vms370841-bib-0006] Cook, M. T. , G. Tzortzis , D. Charalampopoulos , and V. V. Khutoryanskiy . 2012. “Microencapsulation of Probiotics for Gastrointestinal Delivery.” Journal of Controlled Release 162, no. 1: 56–67. 22698940 10.1016/j.jconrel.2012.06.003

[vms370841-bib-0007] Dubey, A. , R. K. Singh , S. Kumari , et al. 2021. “Heat Tolerance of Encapsulated Probiotics During Simulated Pelleting.” Animal Nutrition 7, no. 3: 683–690.

[vms370841-bib-0024] Esmatabadi, M. D. , A. Bozorgmehr , S. N. Hajjari , A. S. Sombolestani , Z. V. Malekshahi , and M. Sadeghizadeh . 2017. “Review of new Insights Into Antimicrobial Agents.” Cellular and Molecular Biology 63, no. 2: 40–48. 10.14715/cmb/2017.63.2.6 28364794

[vms370841-bib-0009] Femia, A. P. , C. Luceri , P. Dolara , et al. 2002. “Antitumorigenic Activity of *Lactobacillus* and *Bifidobacterium* Strains and Their Mixture in Rats Administered a High‐Fat Diet.” International Journal of Cancer 101, no. 5: 519–524.12237891

[vms370841-bib-0010] Gerez, C. L. , M. J. Torres , G. Font de Valdez , and G Rollán . 2019. “Control of Spoilage and Pathogenic Microorganisms by Lactic Acid Bacteria.” Food Control 106: 106710.

[vms370841-bib-0011] Heidebach, T. , P. Först , and U. Kulozik . 2012. “Microencapsulation of Probiotic Cells for Food Applications.” Critical Reviews in Food Science and Nutrition 52, no. 4: 291–311. 22332594 10.1080/10408398.2010.499801

[vms370841-bib-0012] Huang, L. , H. Zhang , X. Liu , et al. 2018. “Characterization of Alginate‐Probiotic Interactions Using FTIR Spectroscopy.” International Journal of Biological Macromolecules 112: 481–490.

[vms370841-bib-0013] Krasaekoopt, W. , B. Bhandari , and H. Deeth . 2004. “The Influence of Coating Materials on Some Properties of Alginate Beads and Survivability of Microencapsulated Probiotic Bacteria.” International Dairy Journal 14, no. 8: 737–743.

[vms370841-bib-0014] Liu, Y. , L. Liu , D. Liu , et al. 2020. “Chitosan‐Gelatin Encapsulation of Yeast for Probiotic Application in Poultry.” Carbohydrate Polymers 240: 116298.

[vms370841-bib-0015] Martins, F. S. , G. Dalmasso , R. M. Arantes , et al. 2009. “ *S. boulardii* Protects the Intestinal Barrier Function and Reduces Inflammation in a Murine Model of Colitis.” Inflammatory Bowel Diseases 15, no. 5: 688–698.

[vms370841-bib-0016] Matulyte, I. , E. Mozuriene , P. Zavistanaviciute , et al. 2021. “Survival and Gastrointestinal Transit of Microencapsulated *Lactobacillus* Strains.” Journal of Dairy Science 104, no. 2: 1102–1114.

[vms370841-bib-0017] Mokarram, R. R. , S. A. Mortazavi , M. B. H. Najafi , and F. Shahidi . 2022. “The Effect of Multi‐Component Matrices on Probiotic Viability.” International Journal of Food Science and Technology 57, no. 4: 1956–1964.

[vms370841-bib-0018] Reque, P. M. , and A. Brandelli . 2020. “Encapsulation of Probiotic Bacteria Using Novel Carriers: A Review.” Critical Reviews in Food Science and Nutrition 60, no. 12: 1993–2002.

[vms370841-bib-0025] Safari, S. , F. Malekvandfard , S. Babashah , A. Alizadehasl , M. Sadeghizadeh , and M. Motavaf . 2016. “Mesenchymal Stem Cell‐Derived Exosomes: A Novel Potential Therapeutic Avenue for Cardiac Regeneration.” Cellular and Molecular Biology 62, no. 7: 66–73. 27453275

[vms370841-bib-0019] Singh, A. , L. Dey , and P. K. Jha . 2023. “Alginate‐Sorbitol Encapsulation Enhances Thermal Stability of Probiotics for Poultry Feed.” Journal of Food Process Engineering 46, no. 2:e14143.

[vms370841-bib-0020] Wang, H. , Y. Wang , X. Zhou , et al. 2019. “Comparative Tolerance of Encapsulated *Lactobacillus* in Simulated Gastrointestinal Conditions.” Lwt Food Science and Technology 101: 71–77.

[vms370841-bib-0021] Yang, S. , S. Wei , Y. Wu , et al. 2024. “Advances in Encapsulation Techniques and Applications in Animal Nutrition.” Food Front 5, no. 3: 1212–1239.

[vms370841-bib-0022] Yang, S. , S. Wei , Y. Wu , et al. 2024. “Encapsulation Techniques, Action Mechanisms, and Evaluation Models of Probiotics: Recent Advances and Future Prospects.” Food Front 5, no. 3: 1212–1239.

[vms370841-bib-0023] Zhang, L. , Y. Zhou , Y. Xie , et al. 2021. “Alginate‐Based Encapsulation Enhances Probiotic Viability and Release Kinetics.” Journal of Functional Foods 87: 104768.

